# Effects of environmental noise on cognitive (dys)functions in schizophrenia: A pilot within-subjects experimental study

**DOI:** 10.1016/j.schres.2016.03.017

**Published:** 2016-05

**Authors:** Bernice Wright, Emmanuelle Peters, Ulrich Ettinger, Elizabeth Kuipers, Veena Kumari

**Affiliations:** a King's College London, Institute of Psychiatry, Psychology and Neuroscience (IoPPN), Department of Psychology, London, UK; b University of Bonn, Department of Psychology, Bonn, Germany; c NIHR Biomedical Research Centre for Mental Health, South London and Maudsley NHS Foundation Trust, London, UK

**Keywords:** Cognition, Performance, Psychosis, Noise management, Urban noise, Social noise

## Abstract

Cognitive impairment, particularly in attention, memory and executive function domains, is commonly present and associated with poor functional outcomes in schizophrenia. In healthy adults, environmental noise adversely affects many cognitive domains, including those known to be compromised in schizophrenia. This pilot study examined whether environmental noise causes further cognitive deterioration in a small sample of people with schizophrenia. Eighteen outpatients with schizophrenia on stable doses of antipsychotics and 18 age and sex-matched healthy participants were assessed on a comprehensive cognitive battery including measures of psychomotor speed, attention, executive functioning, working memory, and verbal learning and memory under three different conditions [quiet: ~ 30 dB(A); urban noise: building site noise, 68–78 dB(A); and social noise: background babble and footsteps from a crowded hall without any discernible words, 68–78 dB(A)], 7–14 days apart, with counter-balanced presentation of noise conditions across participants of both groups. The results showed widespread cognitive impairment in patients under all conditions, and noise-induced impairments of equal magnitude on specific cognitive functions in both groups. Both patient and healthy participant groups showed significant disruption of delayed verbal recall and recognition by urban and social noise, and of working memory by social noise, relative to the quiet condition. Performance under urban and social noise did not differ significantly from each other for any cognitive measure in either group. We conclude that noise has adverse effects on the verbal and working memory domains in schizophrenia patients and healthy participants. This may be particularly problematic for patients as it worsens their pre-existing cognitive deficits.

## 1 Introduction

Cognitive deficits are considered a central feature of schizophrenia (Reichenberg and Harvey, 2007) and predict poor vocational functioning and everyday activities (Reichenberg et al., 2014, Strassnig et al., 2015). While many studies have aimed at potential cognitive improvement, using pharmacological, psychological, or combination methods (Harvey and Bowie, 2012), the removal of influences that may exacerbate existing cognitive deficits in schizophrenia has received relatively less attention. It is possible that environmental factors, such as noise, cause further cognitive impairment in people with schizophrenia (Wright et al., 2014), especially those living in urban environments.

It has long been observed that schizophrenia patients report oversensitivity to sensory stimuli (Bowers and Freeman, 1966) and this has been linked to problems maintaining selective attention (Braff et al., 1977) and screening out irrelevant information (Saccuzzo and Braff, 1981). Previous research has shown that sensory overload, invoked using a combination of excessive auditory and visual stimuli in a controlled environment, causes an increase in manifestations of schizophrenia like behaviour (unusual thought content, social withdrawal, and general cognitive decline) in healthy participants (Gottschalk et al., 1972). A number of studies have already documented the adverse effects of noise, using ‘real-life’ noise stimuli (e.g. multiple conversations, traffic noise), on certain cognitive functions, namely, attention, working memory and episodic recall in healthy adults (Wright et al., 2014). To our knowledge, there is no previous study examining the impact of environmental noise on these cognitive functions in people with schizophrenia.

Therefore, the primary aim of this pilot study was a preliminary investigation into the profile and magnitude of noise effects on cognitive functioning of people with a diagnosis of schizophrenia. Based on the pattern of noise-induced cognitive disruption seen in healthy adults (Wright et al., 2014), we hypothesised that noise would impair the performance of both healthy participants and individuals with schizophrenia on tests of attention, working memory and episodic recall. It was further hypothesised that performance of those with schizophrenia may be more adversely affected than that of healthy participants, given recent observations of increased hemodynamic response (Tregellas et al., 2009) and self-reported sensitivity to noise (Landon et al., in press), in addition to long established sensory gating deficits (Braff, 2010, Patterson et al., 2008), in this clinical population. A secondary aim was to explore possible differential effects of urban (e.g. building site) and social (e.g. bustling shopping centre) noise in schizophrenia patients. Although previous studies have shown similar effects of urban and social noise in cognitive performance of healthy adults (review, Wright et al., 2014), social noise may be relatively more disruptive to cognitive performance of patients, given the association between positive symptoms and exposure to social situations in this population (Freeman et al., 2015).

## 2 Methods

### 2.1 Participants and design

The study involved 18 outpatients who met ICD-10 criteria (World Health Organization, 1992) for diagnosis of schizophrenia and 18 age and sex-matched healthy participants. All participants were assessed on a cognitive battery (*Cognitive assessments*) under three noise conditions (quiet, urban, and social; detailed under *Noise conditions*), with a 1–2 weeks interval between any two assessments. The order of noise conditions (quiet-social-urban, quiet-urban-social, urban-social-quiet, urban-quiet-social, social-quiet-urban, social-urban-quiet) was counter-balanced across participants of both groups (each order used three times per group).

 Patients were recruited from the outpatient clinical services of the South London and Maudsley NHS Foundation Trust and local research registers. Healthy participants were recruited via King's College London circulars to staff and students and local advertisements, and screened to rule out a personal or family history of an Axis I or II disorder. The inclusion criteria required all participants to (i) be aged 18–64, (ii) have normal-to-corrected hearing and vision, (iii) be fluent in English, (iv) have no history of organic brain disorder or primary ICD-10 diagnosis of substance abuse disorder, and (v) have IQ ≥ 80, assessed using the two subtest version of the Wechsler Abbreviated Scale of Intelligence (Wechsler, 1999). An additional exclusion criterion for patients was a period of hospitalisation or a change in medication within 6 months prior to participation.

For sample characterisation purposes (Table 1), all participants were assessed on predicted IQ using the National Adult Reading Test (Nelson and Willison, 1991), handedness using the Edinburgh Handedness Inventory (Oldfield, 1971), subjective sensitivity to noise using the Noise Sensitivity Questionnaire (Schutte et al., 2007), sleep quality using the Pittsburgh Quality of Sleep Inventory (Buysse et al., 1989), and paranoia occurrence using the Paranoia Checklist (Freeman et al., 2005). In addition, symptoms were rated using the Positive and Negative Syndrome Scale (PANSS; Kay et al., 1987) and the age of onset of psychotic symptoms and current medication recorded for those in the patient group.

The study was approved by the NHS Camden and Islington Research Ethics Committee (12/LO/0626). All participants provided written informed consent after the study procedures had been explained to them.

### 2.2 Noise conditions

All sound generating equipment were kept hidden from participants' view in an adjunct sound-proof room, with the connecting door kept open throughout all testing sessions, and speakers (also hidden from participants' view) kept in the sound-proof testing room.

Quiet (No noise): this condition took place in a quiet [~ 30 dB(A)] sound-proof laboratory.

Social noise: the social noise stimulus consisted of background babble and footsteps from a crowded hall [68 dB(A)] with louder peaks of indistinguishable social stimuli superimposed on top [78 dB(A); loud enough to cause annoyance but not damage hearing; Berglund et al., 1999]. No specific words could be discerned from the babble but it mimicked a familiar social environment people encounter in cities.

Urban noise: This noise stimulus consisted of building site noise and did not include any social noise. The noise intensity, time profile, and the number and duration of louder peaks were matched to that presented during the social noise stimulus.

### 2.3 Cognitive assessments

The cognitive battery contained seven tests and included a total of ten measures of psychomotor speed, attention, executive functioning, working memory, and verbal learning and memory (Table 2).

Selection of tests was based on ease and practicality of administration, high test-retest reliability, and lack of practice effects or availability of alternate forms. As detailed in Table 2, alternate test forms for Beads (Dudley et al., 1997), verbal fluency (Benton et al., 1983), and Hopkins Verbal Learning Test-Revised (HVLT-R; Benedict et al., 1998) were used, with each form occurring equally often in the three experimental conditions across participants of both groups. Prior to running this study, a third letter set (T, A, G) to assess verbal phonemic fluency was created and validated against the existing two equivalent 3-letter sets (P, R, W and C, F, L) (Appendix 1). The order of tests in the three experimental conditions was pseudo-randomized across participants, with HVLT-R (Benedict et al., 1998) always in position 1–3 to allow delayed recall testing 25 min later, and the remaining tests presented in a random order. The tasks were presented in the same order during all three sessions for individual participants. The cognitive assessment session lasted approximately 40 min. All sessions were conducted by the same experimenter (BW).

### 2.4 General procedure

Participants were told that the aim of the study was to investigate the effects of stress on cognitive function under ‘real life’ environments. They were requested to abstain from alcohol for at least 24 h prior to the scheduled testing sessions. Smokers (9 patients, 1 healthy participant) were allowed to smoke a cigarette up until 30 min prior to starting the testing session. All sessions began with one or more of the sample characteristic assessments (lasting > 30 min on each occasion) in a quiet environment. Then the experimenter activated the noise stimuli. Following the start of noise exposure, there was a 5-minute (implicit) acclimatization break before cognitive testing commenced to allow participants to get used to the noise. During this break, the experimenter engaged the participants in general conversation. The quiet condition also had a similar 5-minute break before commencing cognitive testing.

### 2.5 Data analysis

**Fig. 1:**
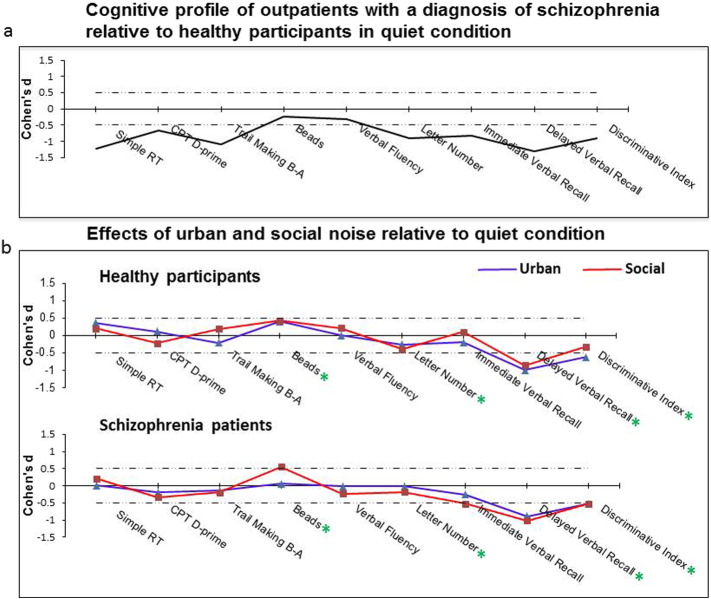
Cognitive profile of schizophrenia patients relative to healthy participants under quiet (1a), and the effects of social and urban noise (1b). For significant noise effects (*, 1b) on the Beads task, a positive value indicates more beads selected (reflecting a suboptimal and indecisive response style) under urban/social noise condition, relative to quiet. For significant noise effects* on all other tests, a negative value indicates reduced performance under urban/social noise condition, relative to quiet.

The schizophrenia and healthy participant groups were compared on sex distribution using χ^2^ and in age, handedness score, IQ, noise sensitivity, sleep quality, and paranoia using independent sample *t*-tests. All cognitive variables were first examined for missing/outlier values, their distribution properties and sphericity. The effects of noise in the two groups were then examined by Group (patients, healthy participants) x Noise Condition (quiet, urban noise, social noise) analysis of variance (ANOVA) with Group as the between-subjects factor and Noise Condition as the within-subjects factor, performed separately for each cognitive variable, followed by lower order ANOVAs and post-hoc mean comparisons as appropriate. Effect sizes for Group and Noise Condition effects were estimated as Cohen's f^2^. Bonferroni correction was applied to the post-hoc analysis of significant noise effects in each measure separately (corrected significance level of p = 0.017 for three pairwise *t*-tests: quiet versus urban, quiet versus social, urban versus social) and Group × Noise interactions (corrected p value of 0.0056 for the nine comparisons: quiet versus urban, quiet versus social, and urban versus social for the two groups separately, and schizophrenia versus healthy participant group for each noise condition separately). Bonferroni correction involving all tests was not applied as the a priori hypotheses planned to consider noise effects in each cognitive domain separately, and pooling all tests would be overly conservative (resulting in a loss of power/increase in Type 2 errors). For a graphical display of group and noise effects (Fig. 1), Z scores for performance of both groups under urban and social noise (standardized using within-group quiet condition) were computed to depict the magnitude of facilitation/disruption caused by the different types of noise.

All analyses were performed using Statistical Package for Social Sciences (version 22). Alpha level for testing significance of effects was maintained at p < 0.05 unless stated otherwise.

## 3 Results

### 3.1 Demographic characteristics

The patient and healthy participant groups were comparable on sex distribution, age, handedness and subjective noise sensitivity levels. Patients, on average, had significantly lower IQ, reduced sleep quality, and higher levels of paranoia, compared to healthy participants (Table 1).

### 3.2 Group differences and the effect of noise on cognitive performance

**Fig. 2:**
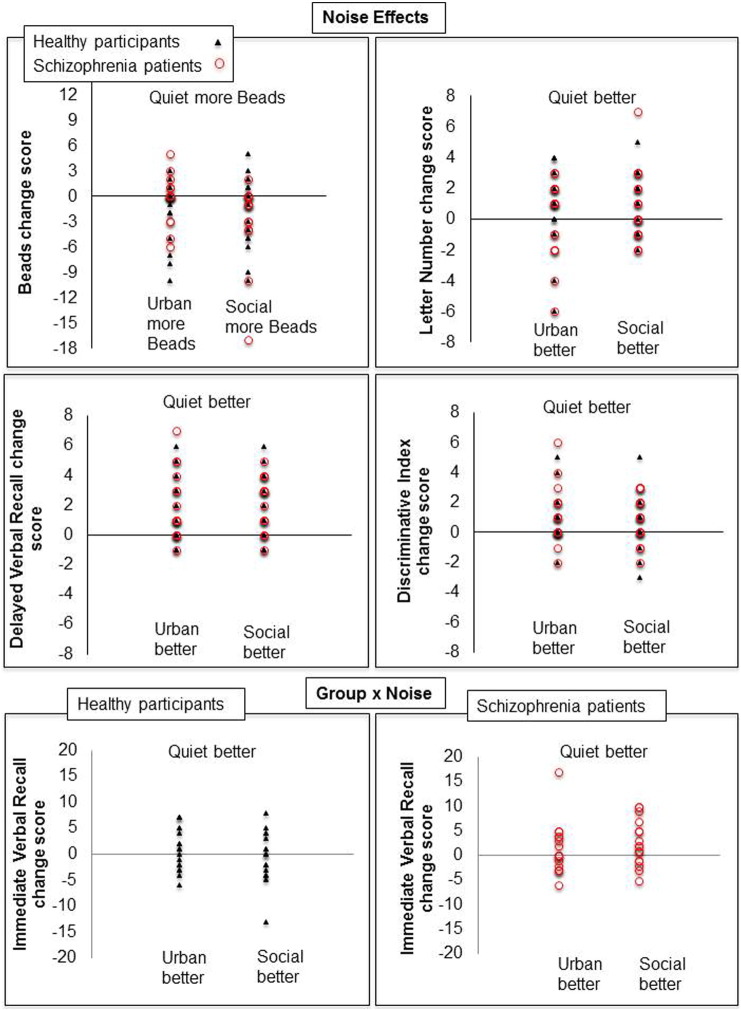
Cognitive change scores (quiet *minus* urban; quiet *minus* social) for variables displaying a significant main effect of noise (top two rows) or a Group × Noise trend for individual patients and healthy participants. For quiet *minus* urban/social noise change scores on the Beads task, a negative value indicates more beads selected, reflecting an indecisive response style, under urban/social condition, relative to quiet. For all other tests, a negative value indicates reduced performance under urban/social noise condition, relative to quiet.

The cognitive profile of patients in the quiet condition, relative to healthy participants, is shown in Fig. 1a. The mean performance scores for both the patient and healthy participant groups under each noise condition are presented in Table 3 and the results of Group × Noise ANOVAs and follow-up analyses are presented in Table 4. The effects of noise on cognitive performance of healthy participants and patients, at the group level, are displayed in Fig. 1b. The cognitive change scores (quiet *minus* urban; quiet *minus* social) for individual patients and healthy participants are displayed in Fig. 2.

*Group effects*: Patient showed impaired performance, relative to healthy participants, across all noise conditions as indicated by significant main effects of Group (Tables 3 and 4) in seven out of ten variables analysed: psychomotor speed (simple reaction time), attention (CPT D-prime), executive function (time to complete TMT Part B *minus* Part A), working memory (Letter Number Scores), immediate and delayed verbal recall, and recognition indices (HVLT-R). Patients' verbal (phonemic) fluency performance was numerically, but not significantly, lower (p = 0.17) than that of healthy participants. The two groups did not differ in the number of beads drawn (Tables 3 and 4; Fig. 1a) or proportion of participants who did/did not jump-to-conclusions (JTC); quiet JTC: patients 27.78%, healthy participants 16.67%, X^2^_1_ = 0.64, p = 0.42; urban noise JTC: patients 33.33%, healthy participants 16.67%, X^2^_1_ = 1.33, p = 0.25; social noise JTC: patients 22.22%, healthy participants 22.22%, X^2^_1_ = 0.00, p = 1.00. We defined JTC as requiring only two beads before making a decision in accordance with previous studies (Garety et al., 2005). The pattern of results remained the same when we defined JTC as requiring only one bead before making a decision following a recent study (Moritz et al., 2015).

*Noise effects*: The main effect of Noise was significant for three out of the ten measures examined: the number of beads drawn (Beads), and delayed verbal recall and recognition (HVLT-R). Relative to the quiet condition, more beads were drawn under social noise, and verbal recall and recognition scores were lower under both social and urban noise conditions (Tables 3 and 4; Fig. 1b). In addition, there was a trend level noise effect in working memory (Letter number scores). Follow-up analysis indicated significant disruption of working memory by social, but not urban, noise relative to the quiet condition.

*Interaction effects*: Group × Noise interaction did not reach formal significance for any of the ten measures examined (Table 4). There was only a trend for a Group × Noise interaction in HVLT immediate recall (Table 4). Follow-up analysis indicated this to be due to lower scores under social noise relative to quiet in patients (p = 0.03; Table 4), with no noise effects on this measure in healthy participants. However, this follow-up analysis did not survive Bonferroni correction (required p ≤ 0.008).

## 4 Discussion

The main findings of this preliminary investigation demonstrated impairments in patients, relative to healthy participants, on most cognitive domains across all conditions, and noise-induced impairments of equal magnitude on specific cognitive functions in both groups. Specifically, we found: (i) significant impairments in psychomotor speed, attention, executive function, working memory, immediate and delayed verbal recall and recognition in patients, relative to healthy participants, across all noise conditions, (ii) significantly reduced delayed verbal recall and recognition under both urban and social noise, and (iii) an indecisive response style on a decision making task (Beads) and significantly reduced working memory, in both groups under social noise relative to the quiet condition. Noise had similar effects in patients and healthy participants on all cognitive measures except (at trend level) verbal immediate recall, which was disrupted (at the uncorrected-significance level) by social noise in patients but not in healthy participants.

Our findings showing a range of cognitive impairment in patients are in line with previous literature (Reichenberg and Harvey, 2007). Our study, however, did not show a significant JTC bias or a significant verbal fluency deficit in patients, most likely because our patient sample was stable with relatively lower scores on relevant symptoms, such as delusions (Garety and Freeman, 2013, Bristow et al., 2014) and we assessed only phonemic fluency in which schizophrenia patients are generally less impaired than semantic fluency (Bokat and Goldberg, 2003).

Noise effects were not apparent across all cognitive domains. However, the significant disruption of delayed recall and recognition by urban as well as social noise, and of working memory by social noise (a non-significant disruption also seen under urban noise), in both groups is in line with our first a priori hypothesis. Social noise also affected performance on the Beads task, with both groups drawing more beads when tested under noise, relative to the quiet condition. At least one participant in each group had drawn the maximum possible number of beads when tested under noise, and this was not the case for any participant when tested in quiet (Table 3). Our results thus suggest a sub-optimal and indecisive response style, rather than an impulsive response style (i.e. fewer beads drawn before making a decision; i.e., increased JTC), under noise. In a recent study (Moritz et al., 2015) that examined the effect of 75 dB building site noise on JTC bias using the Fish Task (a variant of Beads task) in patients with acute delusional symptoms and healthy participants, there was no difference between JTC of patients and healthy participants under the neutral condition but a significant difference emerged during exposure to building site noise. This group difference, however, appears to have been driven mainly by a change in performance of healthy participants, rather than patients, under noise (Fig. 2 in Moritz et al., 2015). The patient group in our study showed the same pattern of noise effects as the healthy group on this (Beads) and other tasks, possibly because our patients had relatively low level of positive symptoms and did not differ from healthy participants in subjective noise sensitivity (Table 1). There was no effect of noise on CPT D-prime. This is in agreement with a previous report of no change in signal detection under noise in healthy adults (Cornblatt et al., 1988). Noise may have stronger adverse effects on other measures of selective attention (Wright et al., 2014). In this study, memory, but not attention and executive functions tasks, showed significant disruption by noise, with delayed recall showing the strongest disruption in both groups (Fig. 1b).

There was little support for our hypothesis of exacerbated noise effects in schizophrenia. Only one measure, immediate verbal recall, that was not significantly affected by noise in healthy participants, showed impairment, at the uncorrected-significance level, in patients under social noise. It remains to be determined whether this finding represents a true effect of small size that would become significant with a larger sample [as per G* power (Faul et al., 2009) analysis, 55 participants per group needed to have 90% power for detecting a significant Noise x Group interaction at p < 0.05], or a chance finding given that the observed effect size for a Group × Noise interaction in other measures was even smaller (Table 4).

There was no evidence of social noise having significantly greater effect than urban noise either in patients or healthy participants. Although some measures were significantly affected only by social noise (and not by urban noise) relative to quiet, performance scores under urban and social noise conditions were not significantly different from each other for any measure in line with previous findings in healthy people (review, Wright et al., 2014). Our findings thus suggest that any association between positive symptoms and social situations in patients (Freeman et al., 2015) did not translate into significantly greater disruption of cognitive performance by social noise, than urban noise. However, the extent to which our ‘social noise’ condition was analogous to a social situation remains unclear.

Despite a lack of significant differential effects of urban or social noise in the patient and healthy participant groups, this pilot study demonstrates that noisy situations may further alter seemingly stable cognitive deficits in people with schizophrenia. Given the association between cognitive function and functional outcomes, noise management, such as reducing exposure to noise where feasible, may improve the lives of people with psychotic disorders. Furthermore, cognitive assessment of clinical groups on noisy wards may lead to over-estimation of cognitive deficits in domains that are sensitive to noise.

Strengths of the present study include the use of a within-subjects design along with the thorough assessment of noise effects in a comprehensive cognitive battery. This design allowed a more coherent interpretation of the effects of noise on different cognitive domains than previous studies (Wright et al., 2014). There are also a number of limitations. Firstly, this pilot study involved only a small number of patients which reduced the power of the study. Secondly, while efforts were taken to counterbalance the presentation of tasks, it is possible that habituation to noise nearer the end of the cognitive battery dampened the magnitude of noise effects, in line with Smith et al.’s (2010) finding of habituation for mental arithmetic after 10 min of noise. Future studies should recruit a much larger sample and also examine the effects of task-related [e.g. presentation order, nature (e.g. verbal versus non-verbal), difficulty level and duration of particular tasks], noise-related (e.g. type, duration and intensity of noise exposure), and participant-related factors (e.g. age, sex, smoking, medication and symptoms in patients) which may directly or indirectly affect the impact of noise on cognitive performance. Finally, the study intervention could not be done double-blind as is generally the case for this sort of design.

In conclusion, the present pilot study found preliminary evidence that noise impairs some cognitive functions in both healthy people and those with schizophrenia. Although there was little evidence that schizophrenia patients are more affected by noise than their healthy counterparts, environmental noise worsened their pre-existing cognitive deficits, particularly in the verbal memory domain. Further studies should focus on finding ways to improve noise management and/or reduce noise exposure, whenever possible.

## Role of funding source

The sponsors had no role in study design; in the collection, analysis and interpretation of data; in the writing of the report; or in the decision to submit the paper for publication.

## Contributors

Bernice Wright, Veena Kumari and Emmanuelle Peters designed the study. Bernice Wright undertook the statistical analysis and prepared the first draft under Veena Kumari and Emmanuelle Peters' supervision. All authors contributed to the final version.

## Conflict of interest

The authors report no biomedical financial interests or potential conflicts of interest.

## Figures and Tables

**Table 1:** Sample characteristics.

Demographic characteristics		Schizophrenia patients (N = 18)	Healthy participants (N = 18)	Test (df)	Statistic	p
Gender (N)	Male/female	10/8	7/11	χ^2^ (1)	1.00	0.32
Age (years)	Mean (SD)	45.50 (7.93)	43.22 (7.97)	t (34)	0.86	0.40
Handedness (EHI) score	Mean (SD)	0.64 (0.59)	0.44 (0.57)^a^	t (30)	0.97	0.34
Pre-morbid IQ (NART)	Mean (SD)	107.82 (10.10)	115.28 (8.23)	t (34)	2.47	0.02
Current IQ (WASI)	Mean (SD)	98.94 (10.92)^b^	114.06 (14.26)	t (32)	3.44	0.002
Noise sensitivity (NoiSeQ)	Mean (SD)	43.61 (12.80)	43.94 (14.11)	t (34)	0.07	0.94
Sleep quality (PSQI total) ↑	Mean (SD)	9.11 (4.03)	4.94 (4.53)^c^	t (33)	2.88	0.007
Paranoia occurrence ↑	Mean (SD)	45.00 (28.68)^c^	2.89 (6.70)	t (33)	6.06	< 0.001

Clinical characteristics (patients only)						
Diagnosis	Schizophrenia only		N (%)	16 (88.89%)		
Schizophrenia with depression		1 (5.56 %)		
Schizophrenia with depression and borderline personality disorder		1 (5.56 %)		
Age at first onset (years)			Mean (SD)	20.44 (11.21)		
Antipsychotic medication	Atypical antipsychotic		N (%)	16 (88.89%)		
Typical antipsychotic		N (%)	2 (11.11%)		
Years in current medications		Mean (SD)	6.63 (7.65)		
PANSS symptoms	Positive		Mean (SD)	19.56 (6.09)		
Negative		Mean (SD)	11.39 (3.45)		
General psychopathology		Mean (SD)	31.61 (11.39)		

EHI = Edinburgh's Handedness Inventory; NART = Nelson Adult Reading Test; WASI: Wechsler Abbreviated Scale of Intelligence; NoiSeQ = Noise Sensitivity Questionnaire; PSQI = Pittsburgh Sleep Quality Index; PANSS = Positive and Negative Syndrome scale.

↑ Higher scores indicate poorer overall sleep quality or greater paranoia levels.

a Reduced N, 3 missing.

b 2 missing.

c 1 missing.

**Table 2:** Details of the cognitive battery.

Cognitive domain	Tests	Dependent variables
Psychomotor speed	Computerised Simple Reaction Time (SRT)	Average RT (ms)
Attention	Continuous Performance Test-Identical Pairs Version (CPT-IP) (Cornblatt et al., 1988)	*D*-Prime (signal detection)
Executive function	Trail Making Test (TMT) (Reitan, 1958)	Time (s) taken to complete Part B *minus* Part A (cost of switching between two tasks)
Beads (60:40 ratio) (Dudley et al., 1997) Alternate forms: red and blue beads; green and black beads; yellow and black beads. Presented under executive function due to evidence of significant associations between Beads performance and working memory and cognitive flexibility (Freeman et al., 2015, Lunt et al., 2012)	Total number of beads selected Jumping to conclusion style (JTC; i.e., proportion making a decision after 2 beads or fewer) (Garety et al., 2005)
Phonemic Verbal Fluency (Benton et al., 1983) Alternate forms: P, R, W; C, F, L; T, A, G	Total correct number of words produced in 60 s (average of three letters)
Working memory	Letter Number Test (Gold et al., 1997)	Total number of correct letter number strings
Verbal learning and Memory	Hopkins Verbal Learning Test – Revised. Alternate forms 1, 2 and 4) (Benedict et al., 1998)	Immediate recall Delayed recall Discriminative index for recognition

**Table 3:** Cognitive performance [mean, standard deviation (SD), range] in patients and healthy participants under quiet, and urban and social noise conditions.

Cognitive domains ^Numbers of patients (s) and healthy participants (h)^	Schizophrenia patients			Healthy participants		
Quiet	Urban	Social	Quiet	Urban	Social
Mean (SD) *Range*	Mean (SD) *Range*	Mean (SD) *Range*	Mean (SD) *Range*	Mean (SD) *Range*	Mean (SD) *Range*
Psychomotor speed						
Simple reaction time (ms) ^s16, h16^	407.89 (114.23)	407.17 (180.58)	382.75 (124.53)	317.80 (72.92)	291.41 (50.77)	303.29 (48.19)
*163.82–582.50*	*227.68–892.37*	*232.88–642.37*	*238.24–567.59*	*233.28–642.01*	*239.02–502.71*

Attention						
CPT: D-prime ^s15, h15^	0.33 (0.21)	0.29 (0.27)	0.26 (0.34)	0.46 (0.17)	0.50 (0.19)	0.42 (0.30)
*− 0.17–0.71*	*− 0.10–0.74*	*− 0.83–0.70*	*0.04–0.65*	*0.12–0.76*	*− 0.62–0.64*

Executive function						
Trail Making Test: time to complete part B minus part A (s)↓ ^s17, h16^	53.92 (25.18)	50.46 (26.64)	49.20 (28.26)	36.00 (16.31)	32.60 (16.22)	39.00 (22.92)
*13.22–86.35*	*7.00–93.44*	*1.09–106.00*	*14.16–71.39*	*13.00–80.05*	*14.00–82.00*
Beads drawn ^s18, h18^	5.50 (4.42)	5.78 (5.12)	7.94 (5.95)	6.50 (4.20)	8.17 (5.33)	8.28 (6.09)
*1.00–14.00*	*1.00–16.00*	*1.00–20.00*	*1.0–14.00*	*1.00–20.00*	*1.00–20.00*
Verbal fluency scores ^s18, h18^	39.28 (11.46)	39.06 (13.18)	36.72 (10.64)	42.22 (9.64)	42.39 (10.97)	44.44 (10.96)
*18.00–60.00*	*21.00–69.00*	*17.00–56.00*	*28.00–59.00*	*23.00–65.00*	*30.00–66.00*

Working memory						
Letter number scores ^s18, h18^	14.17 (4.59)	14.11 (4.48)	13.33 (3.41)	16.83 (2.96)	16.06 (2.96)	15.67 (3.27)
*4.00–22.00*	*2.00–21.00*	*6.00–18.00*	*11.00–22.00*	*12.00–24.00*	*11.00–22.00*

Verbal learning and memory						
HVLT: total immediate recall ^s17, h18^	20.00 (5.02)	18.71 (5.68)	17.41 (3.45)	24.67 (5.75)	23.56 (4.87)	25.28 (4.98)
*11.00–30.00*	*6.00–28.00*	*13.00–24.00*	*13.00–32.00*	*15.00–35.00*	*15.00–32.00*
HVLT: delayed recall ^s17, h17^	6.82 (2.27)	4.82 (2.09)	4.52 (2.27)	9.53 (2.07)	7.47 (3.36)	7.76 (2.88)
*3.00–11.00*	*1.00–10.00*	*1.00–9.00*	*5.0–12.00*	*1.00–12.00*	*3.00–12.00*
HVLT: discrimination index for recognition ^s17, h18^	9.29 (2.14)	8.18 (2.35)	8.18 (2.63)	10.67 (1.53)	9.72 (2.30)	10.17 (1.89)
*4.00–11.00*	*2.00–11.00*	*3.00–2.00*	*7.00–12.00*	*4.00–12.00*	*6.00–12.00*

CPT: Continuous Performance Test; HVLT: Hopkins Verbal Learning Test – Revised.

**Table 4:** The results of the repeated-measures analyses of variance (ANOVA) for cognitive performance.

Cognitive domains ^Numbers of patients (s) and healthy participants (h)^	ANOVA statistics			
Effects (df)	F	p	Effect size f^2^^a^
Psychomotor speed				
Simple reaction time ↓ ^s16, h16^^b^	Group (1, 30)	8.66	**0**.**006**	0.289
Slower simple reaction time in patients.			
Noise (2, 50)	0.63	0.51	0.021
Group × Noise (2, 50)	0.53	0.59	0.017

Attention				
CPT: D-prime ^s15, h15^	Group (1, 28)	5.39	**0**.**03**	0.192
Poorer signal detection in patients.			
Noise (2, 56)	0.70	0.50	0.025
Group × Noise (2, 56)	0.22	0.81	0.008

Executive function				
Trail making test: time to complete part B minus part A ↓ ^s17, h16^	Group (1, 31)	4.70	**0**.**04**	0.152
Patients worse than healthy participants.			
Noise (2, 62)	0.55	0.58	0.017
Group × Noise (2, 62)	0.86	0.43	0.028
Beads drawn ^s18, h18^^c^	Group (1, 34)	0.62	0.44	0.018
Noise (2, 68)	5.30	**0**.**007**	0.156
More beads under social noise than quiet (t = 2.96,df = 35, p = 0.006).			
Group × Noise (2, 68)	1.31	0.28	0.038
Verbal fluency ^s18, h18^	Group (1, 34)	2.00	0.17	0.058
Noise (2, 68)	0.02	0.98	0.001
Group × Noise (2, 68)	1.68	0.20	0.049

Working Memory				
Letter Number Scores ^s18, h18^	Group (1, 34)	4.21	**0**.**05**	0.124
Lower scores in patients.			
Noise (2, 68)	2.96	**0**.**06**	0.087
Lower scores under social noise than quiet (t = 2.96, df = 35, p = 0.006).
Group × Noise (2, 68)	0.38	0.68	0.011

Verbal learning and memory				
HVLT: total immediate recall ^s17, h18^	Group (1, 33)	16.42	**< 0**.**001**	0.497
Patients recalled fewer words.			
Noise (2, 66)	1.31	0.28	0.031
Group × Noise (2, 66)	2.57	***0***.***085***	0.078
Noise effect in patients (2, 32) Lower scores under social noise than quiet (t = 2.36, df = 16, p = 0.03).	2.19	0.13	0.136
Noise effect in healthy participants (2, 34)	1.50	0.24	0.087
Group effect in each noise condition (33) Patients recalled fewer words than healthy people in all conditions: quiet: t = 2.55, p = 0.02; urban noise: t = 2.92, p = 0.006; social noise: t = 5.40, p < 0.001.			
HVLT: delayed recall ^s17, h17^	Group (1, 32)	14.03	**0**.**001**	0.439
Patients recalled fewer words.			
Noise (2, 64)	21.31	**0**.**001**	0.667
Urban (t = 5.36, df = 33, p < 0.001) and social (t = 5.75, df = 34, p < 0.001) noise worse than quiet.			
Group × Noise (2, 64)	0.41	0.67	0.013
HVLT: discrimination index for recognition ^s17, h18^	Group (1, 33)	6.48	**0**.**02**	0.196
Poor word discrimination in patients.			
Noise (2, 66)	6.46	**0**.**003**	0.196
Urban (t = 3.50,df = 34, p = 0.001) and social (t = 2.88, df = 34, p = 0.007) noise worse than quiet.			
Group × Noise (2, 66)	0.56	0.58	0.017

CPT: Continuous Performance Test; HVLT: Hopkins Verbal Learning Test – Revised.

a Cohen f^2^ effect sizes: small = 0.02, medium = 0.15, large = 0.35. Bold p levels indicates significance at < 0.05 or at trend level (p < 0.09).

b Greenhouse-Geisser correction applied as Mauchly's W was significant (p = 0.04).

c The same pattern of results obtained if JTC defined as only requiring one bead following Moritz et al. (2015).

## Appendix A Supplementary data

Development and validation of a third subset (T, A, G) of verbal fluency letters comparable to the existing two sets (P, R, W and C, F, L). Image 1
