# Supplementary material for: Effects of environmental noise on cognitive (dys)functions in schizophrenia: A pilot within-subjects experimental study
Source: Schizophr Res. 2016 May;173(1-2):101–8. doi: 10.1016/j.schres.2016.03.017 (PMC4847736; doi:10.1016/j.schres.2016.03.017)
Supplement: Supplementary file 1 — Development and validation of a third subset (T, A, G) of verbal fluency letters comparable to the existing two sets (P, R, W and C, F, L). [file mmc1.docx]

Appendix A. Development and validation of a third subset (T, A, G) of verbal fluency letters comparable to the existing two sets (P, R, W and C, F, L)

Phonemic VF is commonly used in clinical settings, with good-to-excellent test-retest correlations over 6 or 12 months periods for the same letters (F, A, S) (Basso et al., 1999) or alternative versions (C, F, L and P, W, R). Our noise study, however, required retesting over a much shorter period and three equivalent sets. This led us to create and validate a new 3-letter set (T, A, G). The letters T, A, G and in the new set were chosen because of their equivalent difficulty to the letters in the P, W, R and C, F, L sets (Borkowski et al., 1967). To confirm equivalence of the three 3-letter sets (C, F, L; P, W, R; T, A, G), 38 English speaking healthy participants (20 male, 18 female; 18-64 years old; none included in the noise study) were instructed to generate as many different words as they could in 60 seconds for each of the nine letters (pseudo-randomised presentation across participants; each letter presented 4 times in the same serial position). Perseverations (repetitions) and grammatical errors (changing the ending of a root word) were recorded. The comparability of the three sets was established by a) lack of a significant difference in the sum of words generated to the three letters in each set [mean (SD), C, F, L: 51.92 (10.55); P, W, R: 49.71 (9.61); T, A, G: 50.11 (9.16); repeated measure analysis of variance, F=1.43, df=2,74, p>0.05)], and b) highly significant correlations (p<0.001) between the three 3-letter sets (P, R, W and C, F, L: r =0.75; P, R, W and T, A, G: r=0.79; T, A, G and C, F, L: r=0.80). Very few errors were made for any of the three sets [mean (SD), C, F, L: 1.45 (1.74); P, W, R: 1.42 (1.54); T, A, G: 1.66 (1.83)].

References

Basso, M.R., Bornstein, R.A., Lang, J.M., 1999. Practice effects on commonly used measures of executive function across twelve months. Clin. Neuropsychol. 13, 283-292.

Borkowski, J.G., Benton, A.L., Preen, O., 1967. Word fluency and brain damage. Neuropsychologia 5, 135-140.
